# Risk Assessment of Early Lung Cancer with LDCT and Health Examinations

**DOI:** 10.3390/ijerph19084633

**Published:** 2022-04-12

**Authors:** Hou-Tai Chang, Ping-Huai Wang, Wei-Fang Chen, Chen-Ju Lin

**Affiliations:** 1 Department of Critical Care Medicine, Far Eastern Memorial Hospital, New Taipei 22000, Taiwan; houtai38@saturn.yzu.edu.tw; 2 Department of Industrial Engineering and Management, Yuan Ze University, Taoyuan 32003, Taiwan; s1075416@mail.yzu.edu.tw; 3 Division of Thoracic Medicine, Department of Internal Medicine, Far Eastern Memorial Hospital, New Taipei 22000, Taiwan; pinghuaiwang@gmail.com

**Keywords:** regularized regression, risk prediction, stage I lung cancer

## Abstract

Early detection of lung cancer has a higher likelihood of curative treatment and thus improves survival rate. Low-dose computed tomography (LDCT) screening has been shown to be effective for high-risk individuals in several clinical trials, but has high false positive rates. To evaluate the risk of stage I lung cancer in the general population not limited to smokers, a retrospective study of 133 subjects was conducted in a medical center in Taiwan. Regularized regression was used to build the risk prediction model by using LDCT and health examinations. The proposed model selected seven variables related to nodule morphology, counts and location, and ten variables related to blood tests and medical history, achieving an area under the curve (AUC) value of 0.93. The higher the age, white blood cell count (WBC), blood urea nitrogen (BUN), diabetes, gout, chronic obstructive pulmonary disease (COPD), other cancers, and the presence of spiculation, ground-glass opacity (GGO), and part solid nodules, the higher the risk of lung cancer. Subjects with calcification, solid nodules, nodules in the middle lobes, more nodules, and diseases related to thyroid, liver, and digestive systems were at a lower risk. The selected variables did not indicate causation.

## 1. Introduction

The early symptoms of lung cancer are not obvious, and are easily confused with a cold. Patients are usually diagnosed at an advanced stage when lung cancer is found. According to GLOBOCAN 2020, an online database from the Global Cancer Observatory (GCO) of the World Health Organization’s International Agency for Research on Cancer (IARC), the mortality rate of lung cancer ranked first when both sexes were combined [1]. In 2020, lung cancer accounted for 18% of all cancer deaths worldwide [1]. It was the leading cause of cancer death in men (mortality = 21.5%), second only to breast cancer in women (mortality = 13.7%). In the same year, lung cancer was the most commonly diagnosed cancer in men (incidence = 14.3%), and the third in women (incidence = 8.4%) [1]. The prognosis of lung cancer is poor. Based on the Surveillance, Epidemiology, and End Results (SEER) Program’s Cancer Statistics Review, the 5-year relative survival rate of lung cancer from 2011 through 2017 is 21.7% [2]. Early detection of lung cancer is a topic worthy of further research.

Chest X-ray, computed tomography (CT), and low-dose CT (LDCT) are the most common methods for screening lung cancer. However, these methods may overlook lung cancer due to lesion size, conspicuity, and location [3]. For example, an observational cohort study analyzed 40 instances collected between 1993 and 2001 by six thoracic radiologists at three institutions in the United States [4]. The median diameter of non-small cell lung cancer undetected on a chest X-ray was 1.9 cm. A side-by-side comparison between the chest X-ray and CT scans of missed lung lesions can provide radiologists with causal information on failure detection to improve the interpretation of the plain X-ray [5]. On the other hand, CT and LDCT were reported to be more sensitive to small nodules; the LDCT was able to detect non-calcified lung nodules 10 times more often than the chest X-ray, as studied in [6]. The National Lung Screening Trial (NLST) Research Team found that high-risk participants who underwent LDCT had a 20% reduction in lung cancer mortality compared to those who underwent a chest X-ray in [7]. The Dutch–Belgian NELSON trial [8], the German Lung Cancer Screening Intervention (LUSI) trial [9], and the UK lung cancer screening trial (UKLS) [10] also provided evidence of a mortality reduction by LDCT screening in patients who smoked.

Despite the benefits of LDCT for the early detection of lung cancer pointed out in [11,12], the issues of false positive, overdiagnosis, cost-effectiveness, and radiation exposure of LDCT screening are still of concern [13,14,15,16,17]. The overdiagnosis problem from LDCT may even cause stress and unnecessary treatments to patients [18]. Laboratory tests are potentially to improve the risk assessment of CT screening [19]. This research conducted a retrospective study that used LDCT and health examinations to evaluate the risk of lung cancer.

Early detection and treatment are important to improve the survival rate of lung cancer. This research aimed to construct a risk model of stage I lung cancer for the general population, not limited to high-risk groups who smoke. Clinicians can assess the potential malignancy of lung nodules based on their size, morphology, texture, shape, and distribution by reading LDCT images in practice. However, LDCT images alone may not be sufficient to identify lung cancer in certain circumstances. Considering that LDCT screening is sensitive to detect small nodules but has a high false positive rate, this research used LDCT and health examinations to contribute to the evidence base around overdiagnosis that LDCT may cause. This retrospective study used smoking, the variables from physical examination, personal and family history, routine blood testing, and the variables related to nodule characteristics listed in LDCT reports to develop the risk model.

## 2. Materials and Methods

**Figure 1:**
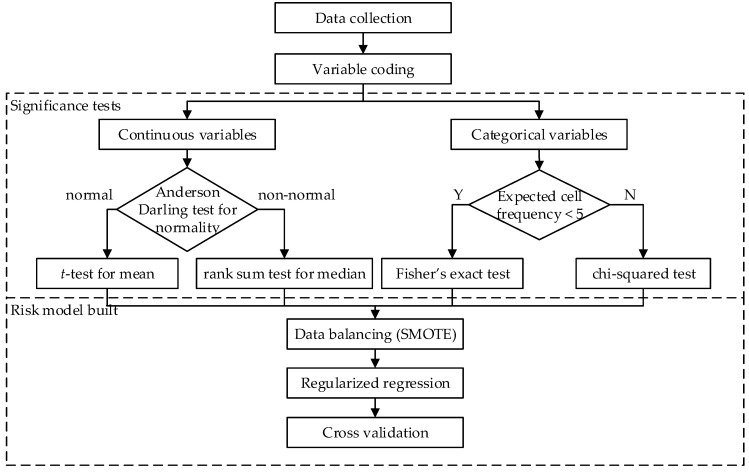
The flowchart of the proposed method.

This research conducted a retrospective study on the medical records collected from a medical center in Taiwan under the Institutional Review Board (IRB) regulation (No. FEMH No: 107065-E). The IRB waived the requirement for informed consent. The research design and methods combined clinical knowledge and statistical analysis. This research used the LDCT and health examinations to predict the risk of stage I lung cancer. The health examinations included physical examinations, smoking record, personal medical history, family history, and routine blood tests. The analysis process contained four parts: data collection, variable coding, significance tests, and risk model built (Figure 1). LDCT can be used for lung cancer screening but not diagnosis. This retrospective study used surgery results as the gold-standard for the diagnosis of stage I lung cancer.

In data collection, the inclusion criteria were to select subjects who had LDCT screening, health examinations, and pathological examination of lung cancer between 2007 and 2017 in the investigated medical center. The exclusion criterion was to exclude the minors under 20 years of age. The data were collected by the first two authors (H.-T.C. and P.-H.W), as the doctors specializing in thoracic medicine at the medical center. The patients who met the selection criteria in the electronic medical record system were selected. The data were de-identified before being passed to the research team for statistical analysis and risk model built. All of the team members received IRB training. The study comprised 133 subjects, 97 in the cancer group and 36 in the non-cancer group. The numbers of the subjects in the two groups were unequal, resulting in imbalanced data. The imbalance ratio of the cancer group to the non-cancer group was 2.69:1. In practice, using LDCT images alone may not be sufficient to determine whether a lung nodule is malignant or benign, especially when the nodule size is small. This study focused on the patients who needed surgery results to confirm tumor malignancy following LDCT detection of lung nodules. Therefore, there were more subjects in the cancer group than in the non-cancer group. Analyzing LDCT images of patients diagnosed with lung cancer can provide clinicians with information on lung cancer identification, thereby reducing unnecessary surgery.

We investigated 40 variables from health examinations and LDCT reports as listed in Table 1 and Table 2. The binary variables with No/Yes were coded as 0/1; female/male as 0/1. The history of several diseases was investigated to study their potential relationship with lung cancer. For example, the variable “COPD” was coded as 1 if any of the following keywords were found in examination reports: chronic obstructive pulmonary disease (COPD), chronic bronchitis, and emphysema. As for LDCT examination, this study used important variables related to nodule counts, size, pattern, and location from the text report provided by radiologists for analysis.

This study performed statistical tests on the significance and independence of variables. Before testing the difference of a continuous variable between the cancer and the non-cancer groups, the Anderson–Darling (AD) test for normality was firstly applied. If data were normally distributed, the t-test was applied to test the difference in mean. Otherwise, the non-parametric rank sum test was applied to test the difference in median. As for assessing the independence of a categorical variable on the variable of groups, the chi-squared test was applied if the expected frequency of the cell in the contingency table was at least five. Otherwise, since the approximation method of the chi-squared test was inappropriate, the Fisher’s exact test was applied to test independence.

The risk model–built phase contained three steps: (1) data balancing, (2) regularized regression, and (3) cross validation. In order to have a better overall prediction performance in the two groups, this study used the synthetic minority over-sampling technique (SMOTE) [20] to balance the sample size of the two groups before applying classifiers. The SMOTE method firstly selects an instance in the minority class, and finds its k nearest neighbors of the same class. Then, a synthetic instance is generated between the selected instance and one of its k neighbors. To overcome the issues of multicollinearity among predictor variables and model over-fitting, this research used the regularized regression analysis to consider both model predictability and interpretability. To avoid obtaining complex models, regularized regression shrinks the coefficient of insignificant variables towards zero by assigning penalty to the magnitude of regression coefficients, as well as the magnitude of error terms. This study compared three classical regularized regression models, ridge regression [21], least absolute shrinkage and selection operator (Lasso) [22], and elastic net [23] to build the prediction model of stage I lung cancer.

Lasso regression imposes an L1-penalty on the regression coefficient, which produces a sparse model by forcing the coefficient of the insignificant variable to zero. The method tends to select one significant variable from a group but skip the other correlated variables. On the other hand, ridge regression imposes an L2-penalty, which makes the coefficients of insignificant variables close to zero. Elastic net can be considered as the combination of Lasso and ridge regression, which imposes both L1 and L2 penalties on the regression coefficients. The objective function is

(1) $$\underset{\beta_{0},\;\beta }{\min}-\left[\frac{1}{n}\overset{n}{\underset{i=1}{\sum }}y_{i}\left(\beta_{0}+x_{i}^{T}\beta \right)-\log\left(1+e^{\left(\beta_{0}+x_{i}^{T}\beta \right)}\right)\right]+\lambda \left[\left(1-\alpha \right)\|\beta {\|}_{2}^{2}/2+\alpha \|\beta {\|}_{1}\right]$$

where the parameter *λ* adjusts the intensity of the penalty term; parameter *α* assigns different weights to L1 and L2 penalties. Elastic net can simultaneously perform variable selection and regularization. Correlated variables are selected in groups if significant by using the method. The elastic net model reduces to Lasso when *α* = 1, and reduces to ridge regression when *α* = 0.

## 3. Results

### 3.1. Summary Statistics

In this retrospective study, 133 subjects were selected according to the inclusion and exclusion criteria from a medical center between 2007 and 2017 in Taiwan. During the time period investigated, only two patients underwent two LDCT examinations. This study selected their first examination results for analysis. One-time records were to avoid having correlated data from multiple visits by the same subject. The count ratio of the cancer group to the non-cancer groups was 2.7:1. The descriptive statistics and the statistical test results are summarized in Table 3 and Table 4.

Among the continuous variables, “Age”, “BMI”, “HGB”, “WBC”, and “Platelet” were normally distributed by the AD test with *p*-values greater than 0.05. The *t*-test was then used to compare the difference of the two groups in mean for these five variables. On the other hand, “Count”, “Diameter”, “BUN“, ”Creatinine”, and “ALT” were not normally distributed by the AD test with *p*-values less than 0.05. The rank sum test was used to test the difference in median. Among these ten continuous variables, only age (*p*-value = 0.026), nodule counts (*p*-value = 0.000+) and diameter (*p*-value = 0.003) are significant variables to lung cancer. The average age (61.33 vs. 56.58) and the median diameter of the maximum nodule (1.67 vs. 1.48) of the cancer group were higher than that of the non-cancer group. The results support that increasing age is a risk factor for lung cancer, and larger nodules are more likely to be cancerous. The median nodule count of the cancer group was lower than that of the non-cancer group (1.57 vs. 3.08). In the result of the routine blood tests, the mean values of blood urea nitrogen, creatinine, alanine aminotransferase, and white blood cell count of the cancer groups were slightly higher than those of the non-cancer group. However, the differences were insignificant.

To test the independence of the variables on groups, the chi-square tests were performed on the variables “Gender”, “Smoke”, “Hypertension”, “Cardiovascular Disease”, “GGO”, “Upper”, “Middle”, and “Lower”; the Fisher’s exact tests were performed on the rest of the 22 categorical variables. Among these categorical variables, only “Spiculated” (*p*-value = 0.000+), “Middle” (*p*-value = 0.002), “Digestive System” (*p*-value = 0.019), and “Solid” (*p*-value = 0.052) were dependent on the group with *p*-values below or around 0.05. The results demonstrated that the LDCT report is informative to determining lung cancer, especially the description of nodule counts, size, and morphology. The non-cancer group had a higher percentage of solid nodules (91.67% vs. 76.29%) or nodules in the middle lobe (30.56% vs. 9.28%) than in the cancer group. On the contrary, spiculated nodules occurred only in the cancer group but not the non-cancer group (0% vs. 29.90%). There were no significant differences between the two groups in the percentages of having a family history of lung cancer and personal medical history. Notably, the non-cancer group had a higher percentage of digested-related diseases, such as colorectal polyp, gastric ulcer (GU), gastroesophageal reflux disease (GERD), and anus polyp, than the cancer group (11.11% vs. 1.03%).

### 3.2. Model Evaluation

**Figure 2:**
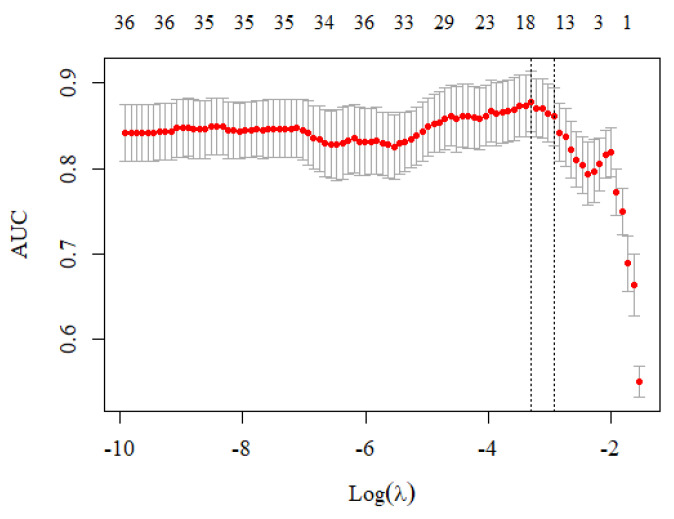
The cross-validation curve at different *λ* values. The left vertical-dotted line with ln *λ* = −3.306 (or *λ* = 0.037) had the highest AUC value, indicating that the model used 17 variables that had the best prediction performance.

**Figure 3:**
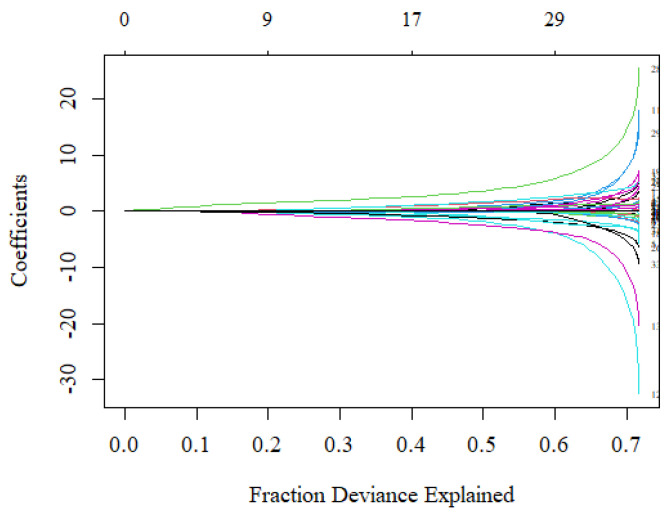
The fraction of deviance explained on the training data when using different numbers of variables. The coefficients of the variables did not differ a lot when selecting 17 variables in the regression model.

The analysis used the smotefamily and glmnet packages in the R language to perform SMOTE and regularized regression. A total of 133 de-identified samples were split at a ratio of 8:2, 106 training data and 27 test data. The analysis applied the SMOTE method to balance the data counts between the cancer and non-cancer groups before constructing the classification models. The cv.glmnet function was used to search for the optimal value of *λ* in each fold based on the AUC criterion. The suggested model used 17 variables where the AUC was maximized at *λ* = 0.037, that was ln *λ* = −3.306 (the left vertical-dotted line in Figure 2). Although using more than 17 variables would increase the fraction of deviance explained, the model would be over-fitting, which can be observed from the large difference in coefficient values (Figure 3). Therefore, the proposed risk model selected 17 out of 40 variables.

The average prediction performance by using the three regularized regression models were similar (Table 5). The best risk prediction model used seven variables from LDCT and ten variables from health examinations to predict the probability of having stage I lung cancer. The regression model is

ln odds = 2.685 × Spiculated + 1.122 × Part Solid + 0.476 × GGO − 0.114 × Count − 0.153 × Solid − 0.324 × Calcified − 0.802 × Middle + 0.997 × Diabetes + 0.383 × Gout + 0.321 × COPD + 0.249 × Other Cancer + 0.100 × WBC + 0.016 × BUN + 0.002 × Age − 0.717 × Thyroid − 1.118 × Liver − 1.733 × Digestive System(2)

**Figure 4:**
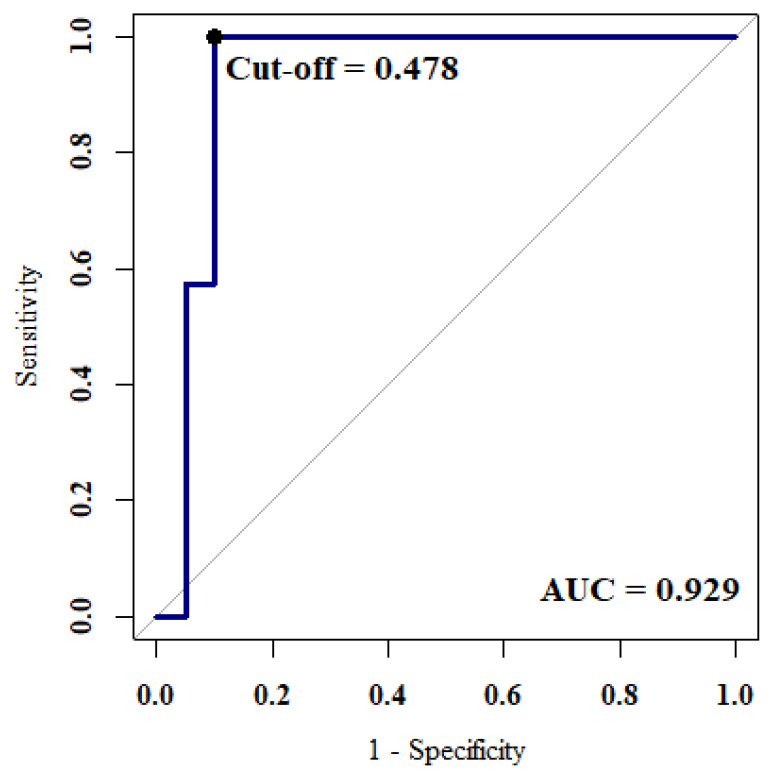
The ROC curve of the best model reached an AUC value of 0.929. The best cut-off value of the proposed regression model was 0.478.

The AUC reached 0.93. The optimal parameter settings were *λ* = 0.037 and *α* = 1, which was a Lasso model. Adding L1 penalty on the regression coefficients shrank the coefficients of the insignificant variables to zero. The best cut-off point was 0.478 where the maximum value of Youden’s index was 0.9 (Figure 4).

## 4. Discussion and Conclusions

Early detection is important to decrease the mortality rate of lung cancer. Although LDCT is known to be sensitive to small nodules in the high-risk group, its false positive rate is high. To effectively detect lung cancer in the early stage, this research used LDCT and health examination data to predict stage I lung cancer. We used and compared the prediction performance of three regularized regression models, Lasso, ridge regression, and elastic net. The best model was the Lasso regression using 17 variables, which had an AUC of 0.93, a sensitivity of 0.85, and an F_1_-measure of 0.92. Lasso regression can handle multicollinearity and perform variable selection by shrinking insignificant coefficients to zero, thereby improving model interpretability.

The result demonstrated that nodule features obtained from LDCT, blood test results, age and disease history were informative to assessing the risk of lung cancer. In the proposed risk model, ten variables had positive coefficients (“Spiculated”, “Part Solid”, “GGO”, “Diabetes”, “Gout”, “COPD”, “Other Cancers”, “WBC”, “BUN”, and “Age”) and seven variables had negative coefficients (“Count”, “Solid”, “Calcified”, “Middle”, “Thyroid”, “Liver”, and “Digestive System”). The model exhibited that the morphology, texture, appearance, and location of nodules were important to evaluate the risk of lung cancer. The coefficient of the variable, “Spiculated“, was the largest in the model (2.685), suggesting that nodules with spiculated borders were highly suspected of malignancy. The finding was consistent with the results in [24]. In addition, the odds of stage I lung cancer were higher in the presence of partial solid nodules or GGO, but lower in the presence of solid nodules or calcification patterns. Similar findings were found in literature [25,26]. Nevertheless, the benign and malignant patterns of calcification should be carefully differentiated, as discussed in [27].

As for the selected variables collected from health examinations, age was known to be the risk factor of lung cancer. In the proposed risk model, a positive coefficient (0.321) for the variable “COPD” indicated a higher risk of lung cancer in the presence of COPD, chronic bronchitis, or emphysema. The phenomenon may be due to smoking being one of the major risk factors for COPD, thereby putting COPD patients at a higher risk of lung cancer. In the study [28], the relative risk of lung cancer for subjects with a previous history of COPD, chronic bronchitis, or emphysema was 2.22, compared with 1.22 for nonsmokers with these lung diseases. Diabetes (coefficient = 0.997) and gout (coefficient = 0.383) are suspected risk factors of lung cancer as mentioned in [29,30,31], although their relationship is not fully understood. This may be due to the fact that patients with diabetes or gout are often associated with obesity and smoking, which are common factors of many cancers. Notably, smoking was not selected in the prediction model. This may be due to the low smoking prevalence in East Asia, especially in females. In the study [32], about one-third of lung cancer patients in East Asia have never smoked. The characteristics of lung cancer in smokers and nonsmokers are different. Frequent epidermal growth factor receptor (EGFR) mutations were observed in the specimens of Asian patients with non-small-cell lung cancer and nonsmokers [33,34].

This study investigated suspicious patients who required surgical confirmation after undergoing LDCT. This was the group of patients whose lung cancers were difficult to distinguish based on LDCT images alone. The findings of this study provided information for evaluating the risk of stage I lung cancer. However, the limitations were the small sample size and potential bias in the selection of subjects. In this study, several diseases were selected as the predictors of lung cancer. However, the selected variables do not necessarily have a causation with lung cancer. The association between these diseases and lung cancer is worthy of further clinical study.

## Figures and Tables

**Table 1:** The 26 investigated variables from health examinations, including physical examinations, medical history, family history of lung cancer, and blood tests. Of these, 18 binary variables were coded as 0/1 for No/Yes or female/male. The other eight continuous variables were from blood test results.

Variable	Coding	Description
Gender	0/1	Female/male
Smoke	0/1	Non-smoking/smoking
PTB	0/1	Tuberculosis (TB), old TB, or tuberculous pleurisy
Lung radiation	0/1	Radiation exposure to lung
Asthma	0/1	Asthma record
COPD	0/1	Chronic obstructive pulmonary disease, chronic bronchitis, or emphysema
Myoma	0/1	Myoma record
Diabetes	0/1	Diabetes record
Hypertension	0/1	Hypertension record
CVA	0/1	Cerebrovascular accident
Gout	0/1	Gout, hyperuricemia
Liver	0/1	Diseases related to liver
Cardiovascular disease	0/1	Diseases related to heart or blood vessels, such as arrhythmia, atrial fibrillation (AF), valvular cardiac valve disease, peripheral arterial occlusive disease (PAOD), and dyslipidemia, and hyperlipidemia.
Digestive system	0/1	Diseases related to digestive system, such as colorectal polyp, gastric ulcer (GU), gastroesophageal reflux disease (GERD), and anus polyp.
Urinary system	0/1	Diseases related to urinary system, such as penile tumors, benign prostatic hyperplasia (BPH), ureteral stone, renal stone, and nephrectomy.
Thyroid	0/1	Diseases related to thyroid, such as thyroid tumor, hypothyroidism, thyroid nodule, thyroidectomy, and goiter.
Other cancer	0/1	Cancer record other than lung cancer
Family lung cancer	0/1	Family history of lung cancer
Age		age at visit (years)
BMI		Body mass index (BMI) (kg/m^2^)
BUN		Blood urea nitrogen (BUN) (mg/dL)
Creatinine		Creatinine (mg/dL)
ALT		Alanine aminotransferase (ALT) (IU/L)
HGB		Hemoglobin (HGB) (g/dL)
WBC		White blood cell count (SBC) (103/μL)
Platelet		Platelet (103/μL)

**Table 2:** The 14 investigated variables from LDCT text reports. Of these, 12 binary variables were coded as 0/1 for No/Yes to describe the presence of nodule pattern, location, and lung condition. The other two continuous variables were nodule count and size.

Variable	Coding	Description
Count		Total nodule counts
Diameter		The diameter of the maximum nodule (cm)
GGO	0/1	Presence of ground-glass opacity (GGO)
Solid	0/1	Presence of solid nodule
Part Solid	0/1	Presence of partial solid nodule
Upper	0/1	Presence of nodule at middle lobe
Middle	0/1	Presence of nodule at upper lobe
Lower	0/1	Presence of nodule at lower lobe
Spiculated	0/1	Presence of spiculation feature
Fibrotic	0/1	Presence of fibrotic pattern
Mosaic	0/1	Presence of mosaic pattern
Calcified	0/1	Presence of calcification pattern
Pneumothorax	0/1	Presence of pneumothorax
Pleural Effusion	0/1	Presence of pleural effusion

**Table 3:** The mean (standard deviation) of the continuous variables in the cancer and non-cancer groups, and the *p*-values of testing the significance of the variables. Age, nodule count, and diameter were found to be significant to lung cancer with *p*-values less than 0.05.

Variable	Non-Cancer	Cancer	*p*-Value
Count	3.08 (2.50)	1.57 (1.39)	0.000
Diameter	1.48 (1.64)	1.67 (0.83)	0.003
Age ^a^	56.58 (9.86)	61.33 (11.11)	0.026
BMI ^a^	24.29 (3.28)	24.19 (3.24)	0.877
BUN	15.41 (4.85)	18.35 (10.36)	0.094
Creatinine	0.93 (0.79)	1.08 (1.50)	0.484
ALT	22.08 (8.92)	23.61 (18.11)	0.639
HGB ^a^	13.32 (1.27)	13.29 (1.66)	0.926
WBC ^a^	6.29 (1.50)	6.61 (1.90)	0.372
Platelet ^a^	221.58 (47.05)	219.8 (50.95)	0.855

^a^ Normally distributed variables by the AD test with *p*-value > 0.05.

**Table 4:** The proportion of the binary variables coded as 1 in the cancer and non-cancer groups, and the *p*-values of the tests for independence. Among these binary variables, having diseases related to the digestive system, nodules in the middle lobe, and spiculated nodules were significant to lung cancer.

Variable	Non-Cancer	Cancer	*p*-Value
Gender ^a^			0.554
Female	58.33	52.58	
Male	41.67	47.42	
Smoke ^a^	33.33	31.96	0.880
PTB	0.00	6.19	0.190
Lung radiation	0.00	1.03	1.000
Asthma	2.78	2.06	1.000
COPD	8.33	16.49	0.278
Myoma	0.00	1.03	1.000
Diabetes	8.33	16.49	0.278
Hypertension ^a^	27.78	38.14	0.266
CVA	0.00	3.09	0.563
Gout	0.00	3.09	0.563
Liver	5.56	1.03	0.178
Cardiovascular disease ^a^	22.22	17.53	0.538
Digestive System	11.11	1.03	0.019
Urinary System	8.33	12.37	0.759
Thyroid	11.11	4.12	0.211
Other Cancer	5.56	14.43	0.233
Family lung cancer	2.78	2.06	1.000
GGO ^a^	27.78	32.99	0.566
Solid	91.67	76.29	0.052
Part Solid	2.78	11.34	0.179
Upper ^a^	80.56	69.07	0.189
Middle ^a^	30.56	9.28	0.002
Lower ^a^	63.89	52.58	0.243
Spiculated	0.00	29.90	0.000
Fibrotic	11.11	15.46	0.781
Mosaic	2.78	1.03	0.470
Calcified	11.11	9.28	0.748
Pneumothorax	0.00	1.03	1.000
Pleural Effusion	2.78	6.19	0.674

^a^ Chi-squared test was applied. Otherwise, Fisher’s exact test was applied.

**Table 5:** The prediction performance of the three regularized regression models. The best model that had the highest AUC value was a Lasso model by using *λ* = 0.037 and *α* = 1.

	Average of 5-Fold Cross Validation			
	Lasso	Ridge	Elastic Net	Best Model (*α* = 1, *λ* = 0.037)
Accuracy	0.72	0.73	0.73	0.89
Sensitivity	0.75	0.78	0.75	0.85
Specificity	0.64	0.58	0.67	1.00
Precision	0.84	0.83	0.86	1.00
F_1_-measure	0.79	0.80	0.80	0.92
G-mean	0.68	0.67	0.70	0.92
AUC	0.78	0.77	0.77	0.93

## Data Availability

Data are available from the Institutional Review Board of the Far Eastern Memorial Hospital for researchers who meet the criteria for the access of confidential data. Requests for the data may be sent to the Institutional Review Board of the Far Eastern Memorial Hospital, New Taipei City, Taiwan (irb@mail.femh.org.tw).
